# Subliminal meaning-contingent attentional orienting: The role of attentional control setting based on displaywide features

**DOI:** 10.3389/fpsyg.2022.1035690

**Published:** 2022-11-17

**Authors:** Huiyuan Wang, Jiajia Yang, Yulin Gao, Ming Zhang

**Affiliations:** ^1^Department of Psychology, Jilin University, Changchun, China; ^2^Faculty of Interdisciplinary Science and Engineering in Health Systems, Okayama University, Okayama, Japan; ^3^Department of Psychology, Suzhou University of Science and Technology, Suzhou, China

**Keywords:** subliminal, meaning contingency, attentional orienting, displaywide features, attentional control setting

## Abstract

People’s subjective factors can affect the spatial allocation of attention, and objects that are more in line with people’s expectations are easier to attract attention. In the current study, we wanted to know whether the meaning-contingent spatial attentional orienting could occur at the subliminal level, that is, whether conscious awareness was needed, and which attentional control settings worked. The current study employed a modified spatial cueing paradigm and the cues were made imperceptible by backward masking. The results showed that the capture effects of the left and the right positions stemmed from the meaning-contingent attentional control setting based on displaywide features, while the inhibition effect of the lower position and the capture effect of the upper position stemmed from the abrupt onset of subliminal cues and their masks. It is concluded that the attentional orienting of meaning contingency could occur at the subliminal level, which was not restricted by conscious perception. In particular, the attentional control setting based on displaywide features played an important role in spatial attentional orienting, which was manifested in the consistent capture effects on the horizontal sides. This study refined and separated the spatial attentional orienting effects, supported the contingent involuntary attentional orienting hypothesis, and expanded its scope of application.

## Preface

Our life is full of a large amount of information. In the face of these complex stimuli, we consciously or unconsciously choose to pay attention to some important events. The question of what kind of stimuli attract attention under what conditions and in what ways is called “attentional capture,” which has always been at the forefront and the focus of research in the field of cognitive psychology. For example, when we want to acquire knowledge, we will pay attention to the books in front of us. This kind of intentional attentional orientation is called top-down, goal-driven and endogenous attentional orientation. However, when some novel or intense stimulus suddenly appears, even if it has nothing to do with our current goal, it will still automatically attract our attention. For example, if we are concentrated on reading, a huge explosion that suddenly takes place outside the window will make us look out of the window involuntarily. This kind of unintentional attentional orientation is called bottom-up, stimulus-driven and exogenous attentional orientation (Posner and Snyder, 1975; Jonides, 1981). In the last 30 years, many psychological researchers have launched a fierce and in-depth debate on the key issue of whether attention capture is a purely bottom-up processing or the result of top-down factor modulation. They considered the impact of consciousness, memory and other factors on attention capture (Luck et al., 2021; Schmid et al., 2021; Theeuwes, 2021), in response to which more integrated views of bottom-up capture and top-down control have gradually formed (Zhang et al., 2021).

A stimulus that is particularly salient in a certain dimension is called a singleton. It is generally believed that the singleton can automatically attract attention in a stimulus-driven way, but the duration of attention will be affected by the current task (Theeuwes, 1991, 2004). If the singleton happens to be the target to be searched or conforms to the current attentional setting, the attention will stay at this position and perform rapid processing; However, when the singleton is a distractor or does not conform to the current attentional setting, the attention will quickly disengage from this position and repoint to the target position, or the signal of giving priority to the singleton is suppressed in a top-down way (Zhang et al., 2021). At the same time, participants will use different search strategies according to the current requirements in the visual search task, and these different search strategies will affect the participants’ responses (Bacon and Egeth, 1994; Wang et al., 2014, 2016). When the task target is a singleton, participants may only search for the stimulus with significant features, regardless of whether it is the target to be searched. In this case, participants adopt the singleton detection mode (SDM), and any singleton may attract attention. However, when there is no singleton in the search range, the participants can only search by relying on the features that define the target. In this case, the feature search mode (FSM) is adopted, and only the stimulus that conforms to the target features is likely to attract attention.

Human subjective factors can affect the spatial distribution of attention. For example, objects that are more in line with human expectations are easier to attract attention. This kind of psychological preparation for attention is called attentional control setting. Involuntary attentional orientation that is affected by the attentional control setting is called contingent attentional orientation (Folk et al., 1992). Only when the contingency between objects such as color, movement and meaning conforms to the current attentional control setting do they affect the transfer and allocation of spatial attention. Attentional control settings can take many forms. The first is based on specific features, that is, only the object (such as red) that is consistent with the target feature (such as red) has the ability to capture attention (Folk et al., 1992, 1994; Goodhew et al., 2014; Huang et al., 2016). The second is the attentional control setting based on non-specific general attributes, that is, all features (such as red and green) under general feature attributes (such as color) can have the ability to affect attention (Folk and Remington, 1998; Folk and Anderson, 2010). The third is the attentional control setting based on displaywide features, which indicate that the task-related target display appears as a whole, and the objects (such as red and green) are consistent with a certain feature (such as the target is red and the non-target is green) in the whole target display (including the target and other objects) and are able to modulate attention (Gibson and Kelsey, 1998; Burnham, 2007).

One of the main paradigms in the study of spatial attentional orientation is the cueing paradigm (Posner and Cohen, 1984; Folk et al., 1992). The two most important stimuli in the paradigm are the cue and the target. The cue appears before the target, and they randomly appear in a certain position in the visual space. The most important factor is the validity of the cue. It focuses on the positional relationship between the cue and the target. If the two appear in the same position, the cue is valid. If the two appear in different positions, the cue is invalid. What researchers need to compare is the reaction time of participants under different cue validity conditions. If the response when the cue is valid is faster than the response when the cue is invalid, then the facilitation or capture effect occurs. However, if the response when the cue is valid is slower than the response when the cue is invalid, then the inhibition effect occurs.

In the early days, the evidence supporting the involuntary contingent attentional orientation hypothesis mainly came from the capture of lower-level feature attributes, that is, the cue and the target were defined by a certain feature (such as color, shape, abrupt onset, etc.). Later, researchers designed a variety of different contingencies between cues and targets, and found that when searching for Chinese character with red meaning, the red cues captured attention, while the same red cues did not lead to attentional capture when searching for Chinese characters without color meaning (Wang et al., 2014). A study in the same period examined the capture effect of red and green cues when the target was word “RED” and “GREEN” respectively. It was found that only color cues consistent with the target semantics could capture attention, while when the target meaning changed, the capture ability of color cues was reversed (Goodhew et al., 2014). In addition, under the condition that the cue was a Chinese character with color meaning and the target was a color feature, we also found that only the cue consistent with the target semantics had the ability to modulate the distribution of attention (Wang et al., 2016, 2018). These results indicated that the involuntary shifting of attention in visual space can occur not only at the level of perceptual features, but also at the higher level of semantic concepts.

A recent study has adopted the cueing paradigm to establish the meaningful contingency between cues and targets, observed the attentional orientation effect of cues in different spatial positions, and investigated the modulation of meaningful contingency between objects on spatial attentional orientation (Wang et al., 2021). The results showed that the meaning-contingent attentional orientation showed a change from the inhibition effect to the capture effect from the lower field to the upper field, and the degree of attentional orientation effect was affected by the nature of guiding cues. The research of Wang et al. (2021) supports the contingent attentional orientation hypothesis proposed by Folk et al. (1992) and provides new evidence for meaning-contingent attentional orientation, that is, the involuntary orientation of cues is determined by whether the cues have task-related attributes or meaning concepts. Only objects with task-related attributes or concepts that conform to the current attentional control setting can attract attention and lead to involuntary attentional transfer to their positions.

Although there is much evidence that, the perceptual and meaningful contingency between objects can modulate the attention to cues under the condition of the cue being perceptible (Folk et al., 1992; Goodhew et al., 2014; Huang et al., 2016; Wang et al., 2018), the question whether the visual spatial attentional orientation must involve consciousness needs to be discussed more explicitly. Some studies have shown that when cues are imperceptible, the perceptual contingency between objects can modulate spatial attentional orientation. For example, subliminal color cues can trigger the contingent attentional capture due to the influence of the attentional control setting caused by the current task goal (Ansorge et al., 2009, 2010). However, the issue of whether the meaning-contingent attentional orientation requires conscious participation has not been discussed in depth. In addition, Wang et al. (2021) posit that the attentional orientation effect in their experiment is affected by the top-down attentional control setting, but the attentional control setting may come from two types. For color targets, the first is the attentional control setting based on non-specific general attributes. Because the target is a color attribute (red or green), the Chinese characters with color meaning are all contingent on the target and have the ability to modulate attention; the second is the attentional control setting based on the displaywide features. Because there are both red and green in the target display, the Chinese characters with the corresponding color meaning have meaning contingency with the whole target display and can modulate attention. Although the experimental results can be interpreted as the attentional orientation influenced by top-down factors regardless of what kind of attentional control setting it is based on, researchers have not ascertained the types and functions of the attentional control settings. To sum up, the purpose of this study is to solve: (1) whether the meaning-contingent spatial attentional orientation needs conscious participation, that is, whether the subliminal cues masking meaning trigger the meaning-contingent attentional orientation; and (2) what kind of attentional control setting causes subliminal meaning-contingent attentional orientation.

## Pre-experiment: Determination of subliminal cues

The purpose of the pre-experiment is to determine that the cues in this study are subliminal, and their meaning cannot be perceived. The participants are required to identify the identities of the cues through backward masking combined with the subjective reports of the participants. The total presentation time of cues and their masks is the same as that of supraliminal cues by Wang et al. (2021). The experimental hypothesis is that if the cue meets the subliminal requirement and its meaning cannot be perceived, the correct response to the cue is at the chance level, that is, 50%; if the accuracy exceeds 50%, the experimental requirements are not met. At the same time, the cue location was also used as an independent variable to investigate whether the cue’s perceptibility was affected by its location.

### Method

#### Participants

Thirty-two undergraduate students participated in the experiment (26 females and 6 males^1^), with an average age of 21.06 ± 1.22 years (19–23 years). One of them was left-handed and the others were right-handed. All participants’ mother tongue was Chinese, they had not participated in similar experiments, and they had normal or corrected visual acuity without color blindness or color weakness. Before the experiments, G * Power software (Faul et al., 2007) was used to estimate the sample size. We designed the effect size f to be 0.25, the α err prob. to be 0.05, and the Power (1-β Err prob) to be 0.95. The calculated sample size of the pre-experiment was 23, therefore, the amount of participants is sufficient.

#### Experimental instruments and materials

**Figure 1:**
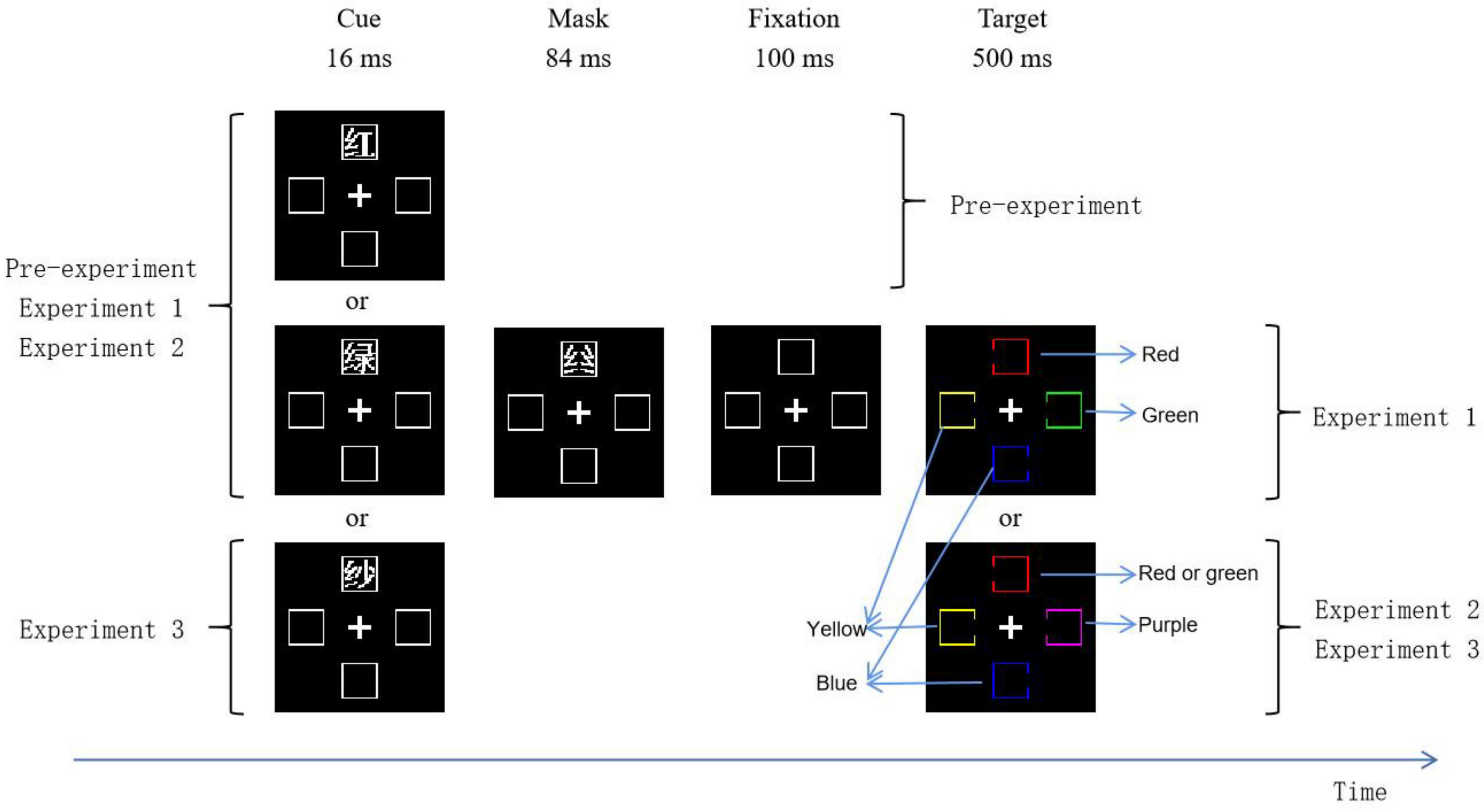
Experimental flow chart in this study.

The 18.5-inch color display was used for presenting stimuli, the display resolution was 1,366 × 768, and the refresh frequency was 60 Hz. The experimental program was compiled by E-Prime and ran on the Windows operating system. The display background of the experiment was black, and the fixation display, cue display and mask display were presented according to the design (see Figure 1). The fixation display included the central fixation plus (white, 0.48 ° × 0.48 °) and the upper, lower, left and right boxes (gray, 1.53 ° × 1.53 °), with the center of the box located 4.4° from the center of the display. A white “红” (Chinese character meaning red) or “绿” (Chinese character meaning green) appeared in a box shaping cue display, and the white Chinese characters were cues. Then, The Chinese character cue was replaced by the symbol to form a mask display. The symbol was the mask, and the mask and the cue always appeared in the same position.

#### Experimental procedure and design

Participants sat in a comfortable chair, keeping a horizontal distance of 63 cm between their eyes and the center of the monitor, and instructions were presented to participants to ensure they understood the experimental process and requirements. In one trial, stimuli were presented in the order of fixation display (500 ms), cue display (16 ms), mask display (84 ms) and fixation display (100 ms). The total time from the beginning of the cue to the end of the second fixation was 200 ms. It is generally believed that eye movements to the cue and other positions will not occur in such a short time (Colgate et al., 1973). The participants understood the arrangement of the experiment. Their task was to judge whether the cue that appeared in the current trial was “红” or “绿” and press the keys with both hands as quickly and accurately as possible. Half the participants were instructed to press the “Z” key with the index finger of the left hand when the Chinese character “红” appears; when the Chinese character “绿” appears, they were instructed to press the “/” key with the index finger of the right hand. The other half of the participants were instructed to do the opposite. After the participants pressed the key, the trial ended. There was a random blank display from 1,400 ms to 1,600 ms between two trials.

A 2 × 4 repeated measurement design was used. The first independent variable was cue identity, including two levels of “红” and “绿.” The second independent variable was the cue location, including four levels: up, down, left and right. Each participant completed 8 practice trials and 96 formal trials. The formal trials were devided into three parts with breaks under the participants’ control. After the experiment, participants were asked whether they could perceive and distinguish cue stimuli. The experiment lasted about 8 min.

### Results and analysis

**Figure 2:**
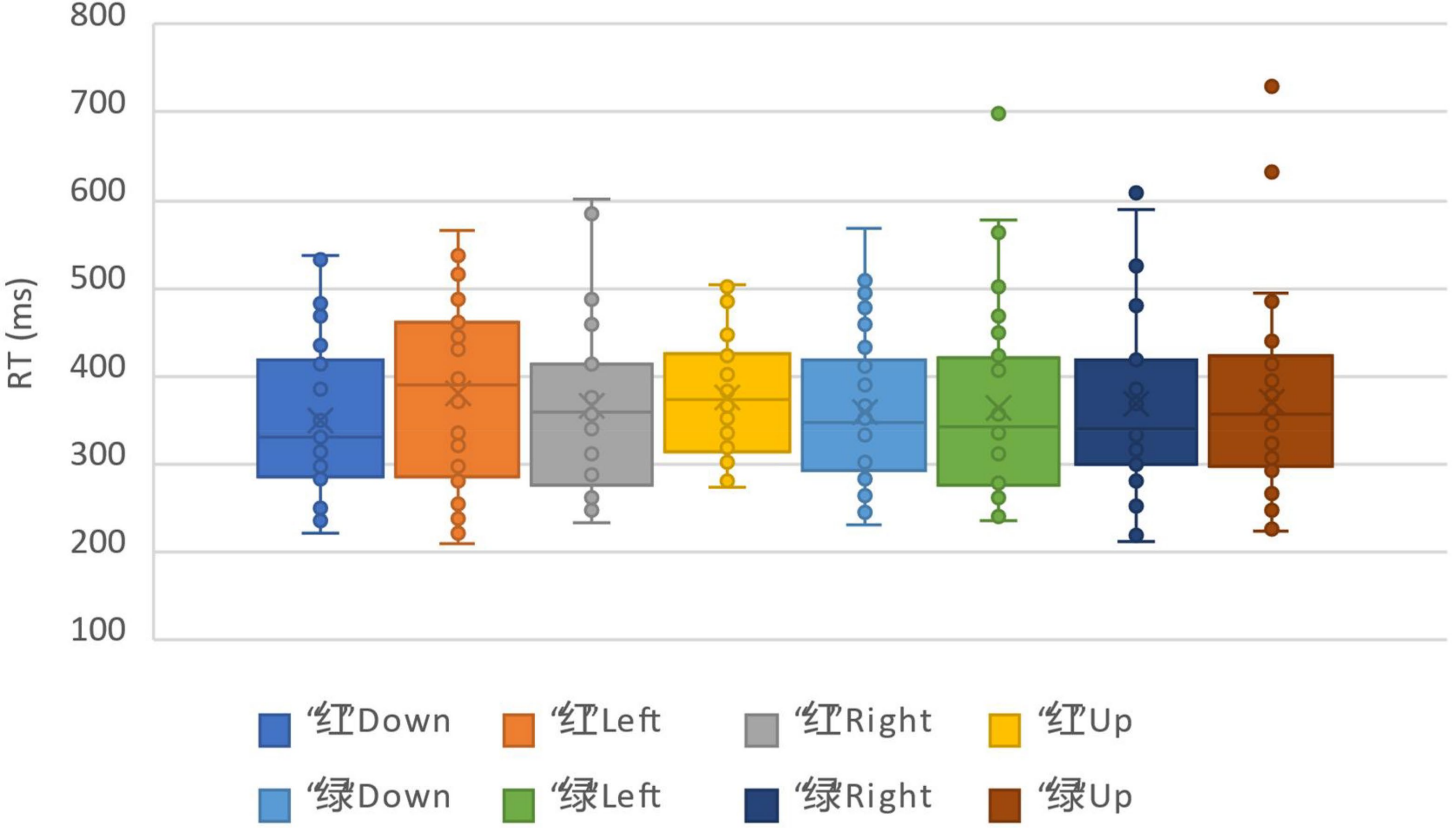
The data distribution of all participants under all conditions in pre-experiment.

For the analysis of the correct reaction times (RTs), 48.75% of the data were deleted by deleting the records of incorrect reactions and the records whose reaction times were outside the three standard deviations above and below the average. The data distribution of all participants under all conditions was shown in Figure 2. We calculated the average reaction times and accuracy rates under the combinations of each cue identity and cue location (Table 1). The repeated measure ANOVA of response time showed that the main effects of the two factors were not significant, respectively: cue identity, *F*(1,31) = 0.08, *p* = 0.77; and cue location, *F*(3,93) = 1.29, *p* = 0.28. The interaction of the two factors was not significant, *F*(3,93) = 0.32, *p* = 0.81. This indicated that the two cues had the same effect on reaction time, which was not affected by the location of the cues.

**Table 1:** Average reaction times and accuracy rates under the combinations of the cue identity and the cue location in the pre-experiment (M ± SD).

	Cue identity	Cue location			
		Down	Left	Right	Up
RT (ms)	“红”	350.15 ± 84.15	380.70 ± 101.22	366.76 ± 100.73	376.59 ± 67.28
	“绿”	359.40 ± 83.06	364.17 ± 110.50	367.40 ± 107.02	371.26 ± 111.96
ACC (%)	“红”	46.16 ± 22.31	52.66 ± 23.75	48.28 ± 33.20	54.06 ± 24.09
	“绿”	51.91 ± 25.17	53.69 ± 25.85	50.84 ± 29.65	49.72 ± 22.95

In addition, the repeated measurement ANOVA of the accuracy (ACC) showed that the main effects of the two factors were not significant: cue identity, *F*(1,31) = 0.06, *p* = 0.81; and cue location, *F*(3,93) = 0.78, *p* = 0.51. The interaction of the two factors was not significant, *F*(3,93) = 0.36, *p* = 0.78. There was no significant difference between the accuracy of the 8 conditions and that of the one-sample *t* test with 50%, |*t|*s ≤ 0.97, *p*s ≥ 0.34. When the cue location factor was not considered, the correct rate of the “红” cue was 50.81 ± 17.00%, and that of the “绿” cue was 51.69 ± 14.79%, which were not significantly different from 50%, *t*s ≤ 0.65, *p*s ≥ 0.52. It could be seen that the forced selection of cues was at a chance level regardless of whether the cue location factor was considered or not. At the same time, participants reported subjectively that they were unable to perceive or identify the cues.

### Discussion

The pre-experiment investigated the participants’ perception of cues by manipulating the identity and location of the cues. The results showed that the correct response rates under all experimental conditions were by chance and were not affected by cue identity or cue location. At the same time, participants’ subjective reports failed to detect the masked cues. The pre-experiment results showed that the cues reached the subliminal level through masking, which met the requirements for cues in this study and could be used as materials in the follow-up study.

## Experiment 1: Subliminal meaning-contingent attentional orientation

This experiment investigates the impact on attentional choice of Chinese character cues which are not perceptible but whose meaning is contingent on the targets when people are looking for something with color features, or whether the meaning-contingent attentional orientation needs perceptual participation. It uses white Chinese characters “红” and “绿” with color meaning as cues for cueing and backward masking and red and green boxes as targets. To avoid the participants adopting the SDM in the experiment, the objects in the target display are different colors, and the target will not become a singleton. The participants search according to the defined color features of the targets. In order to compare the results of the supraliminal meaning-contingent attentional orientation, the experimental design refers to the research of Wang et al. (2021). The experimental hypothesis is that if there is a certain attentional orientation effect, the contingent attentional orientation at the meaning level can reach the subliminal level without the participation of perception.

### Method

#### Participants

Thirty-one undergraduate students (17 females and 14 males) participated in the experiment, with an average age of 22.23 ± 1.16 years (19–24 years). All were right-handed. Other characteristics are the same as those of the pre-experiment. The calculated sample size of the pre-experiment used by G * Power was 15, therefore, the amount of participants is sufficient.

#### Experimental instruments, materials and procedures

Experiment 1 added the target display compared with the pre-experiment, which appeared after the second fixation display. All boxes of the target display were randomly changed into red (REB: 255 0 0), green (RGB: 0255 0), yellow (RGB: 255255 0) and blue (REB: 0 0255). At the same time, each box had a gap on the left or right, of which two gaps were on the left and two gaps were on the right. The presentation time of the target display was 500 ms. The red or green box was the target (see Figure 1). Due to the random location and the lack of predictability of the cues, participants have no reason to actively pay attention to cues and masks. Participants understood the arrangement of all stimulation displays. Their task was to determine the position of the gap in the target box; they used both hands to press buttons as quickly and accurately as possible. If the gap is on the left, they pressed the “Z” key with the index finger of the left hand; and if the gap is on the right, they pressed the “/” key with the index finger of the right hand. The other instructions and characteristics are the same as those of the pre-experiment.

#### Experimental design

A 2 × 2 × 4 repeated measurement design was used. The first independent variable was cue validity, including two levels: valid cue (cue and target appeared in the same position) and invalid cue (cue and target appeared in different positions). The second independent variable was the cue-target semantic congruency, including semantic congruency and semantic incongruency. The “红” cue-the red target and the “绿” cue-the green target were semantic congruent, while the “红” cue-the green target and the “绿” cue-the red target were semantic incongruent. The third independent variable was the cue location, including up, down, left and right levels. The congruency between stimuli adopts the Block design, that was, the identity of the cue and the feature of the target in each block were fixed. Therefore, in each trial, the participants knew which Chinese character the cue was and which color the target was. The order of the blocks was random. Each block includes 12 practice trials and 128 formal trials. The formal trials were divided into four parts with breaks under the participants’ control. The experiment lasted about 40 min.

### Results and analysis

**Figure 3:**
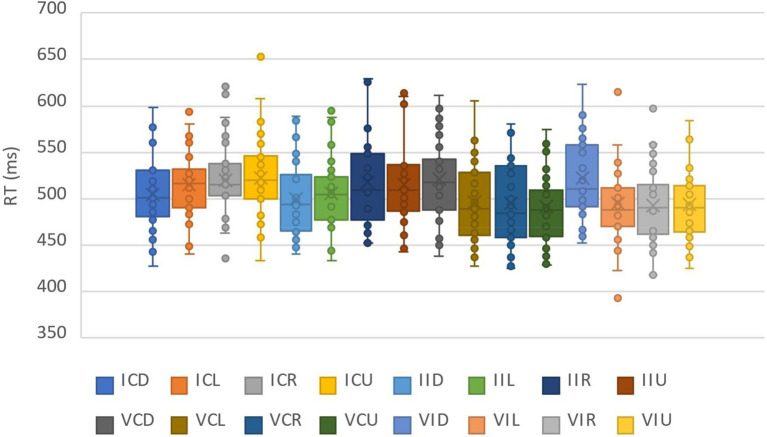
The data distribution of all participants under all conditions in Experiment 1. In the legend, the first capital letter represents the validity of the cue, I represents the invalidity of the cue, and V represents the validity of the cue. The second capital letter represents the semantic congruency of the cue and the target, C represents congruency, and I represents incongruency. The third capital letter represents the position of the cue, D represents down, L represents left, R represents right, and U represents up.

**Figure 4:**
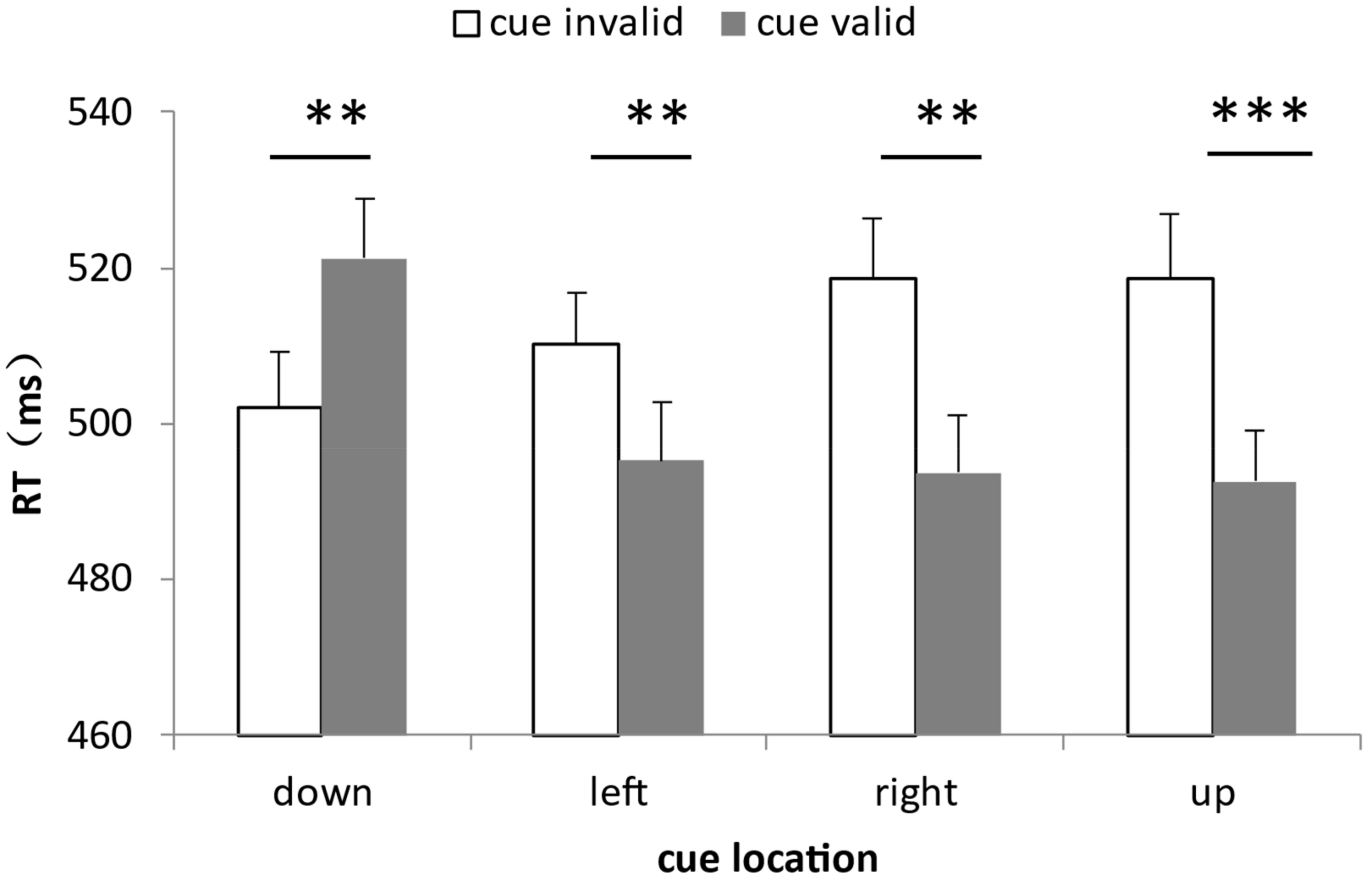
Interaction between cue validity and cue location in Experiment 1. When the cue appeared in the lower position, the response when the cue was invalid was faster than that when the cue was valid; when the cue appeared in other places, the response when the cue was invalid was slower than when the cue was valid (*p < 0.05, **p < 0.01, ***p < 0.001, the same below).

For the analysis of the correct RTs, a total of 5.39% of the data were deleted by deleting the records of incorrect reactions and the records of those reaction times outside the three standard deviations above and below the average. The data distribution of all participants under all conditions was shown in Figure 3. We calculated the average response times and accuracy rates under the combinations of the cue validity, the cue location and the cue-target semantic consistency (Table 2). The repeated measure ANOVA of response time showed that the main effect of cue validity was significant, *F*(1,30) = 8.89, *η_p_^2^* = 0.23, *p* = 0.006, with the response of the target at the cue location (500.72 ms) being generally faster than that of the target at the non-cue location (512.39 ms). The main effect of the cue location was significant, *F*(3,90) = 7.84, *η_p_^2^* = 0.21, *p* < 0.001. Multiple comparisons showed that the response of the lower position was slower than that of other positions, *p*s ≤ 0.002, and there was no difference among other positions, *p*s ≥ 0.12. The main effect of the cue-target semantic congruency was not significant, *F*(1,30) = 0.62, *p* = 0.45. The interaction of the three factors was not significant, *F*(3,90) = 0.36, *p* = 0.78. More importantly, the interaction between cue validity and cue location was significant, *F*(3,90) = 23.46, *η_p_^2^* = 0.44, *p* < 0.001. The simple effect test showed that when the cue appeared in the lower position, the response when the cue was invalid (502.22 ms) was faster than that when the cue was valid (521.30 ms), *F*(1,30) = 12.87, *η_p_^2^* = 0.30, *p* = 0.001. When the cue appeared in other positions, the response when the cue was invalid was slower than that when the cue was valid to different extents, which were respectively: left position, 510.15 ms when the cue was invalid, 495.35 ms when the cue was valid, *F*(1,30) = 10.94, *η_p_^2^* = 0.23, *p* = 0.002 right position, 518.53 ms when the cue was invalid, 493.67 ms when the cue was valid, *F*(1,30) = 14.53, *η_p_^2^* = 0.33, *p* = 0.001 and upper position, 518.67 ms when the cue was invalid, 492.57 ms when the cue was valid, *F*(1,30) = 24.83, *η_p_^2^* = 0.45, *p* < 0.001 (see Figure 4). The other two-factor interactions were not significant. They were the interaction between semantic congruency and cue validity, *F*(1,30) = 3.95, *p* = 0.06 and the interaction between semantic congruency and cue location, *F*(3,90) = 0.67, *p* = 0.58. At the same time, we also analyzed the interactions among the target position and other factors, and found that there were no two-factor or three-factor interactions among the target position, semantic congruency and cue location, *F*s ≤ 1.62, *p*s ≥ 0.11.

**Table 2:** The average response times and accuracy rates under the combinations of cue validity, cue-target semantic congruency and cue location in Experiment 1 (M ± SD).

	Congruency	Cue validity	Cue location			
			Down	Left	Right	Up
RT (ms)	Congruent	Invalid	504.68 ± 40.00	514.59 ± 36.80	520.03 ± 45.34	521.78 ± 47.33
		Valid	520.55 ± 43.74	496.35 ± 43.29	494.44 ± 44.47	490.92 ± 37.69
	Incongruent	Invalid	499.76 ± 41.93	505.72 ± 40.43	517.03 ± 46.29	515.55 ± 46.58
		Valid	522.04 ± 45.24	494.36 ± 46.78	492.91 ± 43.70	494.21 ± 38.65
ACC (%)	Congruent	Invalid	94.90 ± 5.80	95.97 ± 4.98	93.61 ± 6.23	92.77 ± 6.95
		Valid	95.19 ± 7.91	92.52 ± 9.02	93.81 ± 9.25	98.55 ± 3.16
	Incongruent	Invalid	95.10 ± 4.37	94.65 ± 6.71	94.74 ± 4.74	93.19 ± 6.33
		Valid	95.00 ± 7.26	95.23 ± 6.56	94.97 ± 5.28	98.06 ± 2.85

In addition, the repeated measurement ANOVA of accuracy showed that the main effects of the three factors were not significant, which were: cue validity, *F*(1,30) = 3.63, *p* = 0.07; cue location, *F*(3,90) = 2.04, *p* = 0.12; and cue-target semantic congruency, *F*(1,30) = 0.58, *p* = 0.45. The interaction of the three factors was not significant, *F*(3,90) = 1.59, *p* = 0.20. The interaction between cue validity and cue location was significant, *F*(3,90) = 10.05, *η_p_^2^* = 0.25, *p* < 0.001. The simple effect test showed that when the cue appeared in the upper position, the correct rate when the cue was invalid (92.98%) was lower than that when the cue was valid (98.31%), *F*(1,30) = 45.02, *η_p_^2^* = 0.60, *p* < 0.001. When the cue appeared in other places, there was no difference between the correct rate when the cue was invalid and the correct rate when the cue was valid, *F*s ≤ 1.72, *p*s ≥ 0.20. The other two-factor interactions were not significant. They were the interaction between semantic congruency and cue validity, *F*(1,30) = 0.52, *p* = 0.48 and the interaction between semantic congruency and cue location, *F*(3,90) = 0.60, *p* = 0.62. Through the calculation of G*power software, the statistical powers of the significant results were greater than 0.95. At the same time, participants reported subjectively that they were unable to perceive or identify the cues.

### Discussion

In Experiment 1, by manipulating cue validity, cue-target semantic congruency and cue location, the modulation of subliminal Chinese character cues whose meaning was contingent on the target color on spatial attentional selection was investigated. The results showed that when the subliminal cue appeared in the lower position, it triggered an inhibitory effect, indicating that this region was inhibited, which corresponded to the slower response of the lower position in the experiment. When the subliminal cue appeared in other positions, it triggered capture effects of different degrees; that was, these positions got more attention, and when the target appeared in these positions, it underwent faster processing, which also corresponded to the faster response of the upper position in this experiment. In addition, the paired sample t test showed that there was no significant difference between the two when the subliminal cue appeared in the left and right positions, *t*(30) = −1.59, *p* = 0.12, which showed that the subliminal cue had the same capture degree in the horizontal positions and did not exhibit differences between the left and right visual fields.

The results of Experiment 1 showed that subliminal Chinese character cues meaning-contingent on the color targets could modulate spatial attentional allocation, which supported the contingent attentional orientation hypothesis. It also pointed out that the contingent spatial attentional orientation could occur not only at the level of abstract meaning, but also at the subliminal level, without the participation of consciousness. In addition, Experiment 1 found the changing trend of spatial attentional orientation in vertical space, which was consistent with the effect of meaning-contingent attentional orientation triggered by supraliminal cues (Wang et al., 2021). In particular, in the same Chinese character cue-color target mode, in terms of the difference in the attentional orientation effect between the upper and lower positions, the difference in Experiment 1 was 26.10- (−19.08) =45.18 ms, which was equivalent to the difference in Experiment 2 (38.06 ms) of Wang et al. (2021), showing no significant difference whether consciousness was involved or not.

In addition, in Experiment 1, no interactions between the cue-target semantic congruency and other factors were found; that was, under all conditions, whether the semantics of cue and target were congruent or not, the effects caused by the subliminal cue were consistent, which was still in line with the results of Wang et al. (2021). There may be two explanations for this result. The first is that participants adopt a general attribute-based attentional control setting; that is, stimuli that conform to the general attribute have the possibility of modulating attention. In Experiment 1, the target was red or green, and the participants may hold the attentional control setting of looking for “color.” Under the attribute of “color,” the Chinese characters “红” and “绿” conform to the meaning-contingent attentional control setting and have the same ability to modulate attention. The second explanation is that the participants adopted an attentional control setting based on the displaywide features; that is, the objects contingent on any element of the target display can modulate the spatial attention. In Experiment 1, there are both red and green elements in the target display; that is, when one color is the target, another color also appears as a distractor, forming a part of the whole target display. Then, in this set of attentional control setting based on the displaywide features, the Chinese characters “红” and “绿” may both be meaning-contingent on the target display and acquire the ability to modulate the spatial attention.

## Experiment 2: The attentional orientation excluding the attentional control setting based on the displaywide features

In order to separate the above two kinds of attentional control settings based on general attributes and displaywide features in subliminal meaning-contingent attentional orientation, Experiment 2 excluded the possibility of the latter by adjusting the composition of the target display. The effects of Experiment 1 and Experiment 2 were then compared to investigate the role of attentional control setting based on displaywide features in subliminal meaning-contingent attentional orientation. The specific operation is that when one of the red and green colors is used as a target, the other color will no longer appear as a distractor in the target display, but a fixed color will be selected instead. In this way, except for the target, the cue is no longer contingent on other objects of the target display. The experimental hypothesis is that if the results of Experiment 1 and Experiment 2 are consistent, at least in this study, the attentional control setting based on the displaywide features does not play a role in subliminal meaning-contingent attentional orientation; If the results of the two experiments are different, it indicates that the attentional control setting based on the displaywide feature has an effect and the effect is the difference between the two experiments.

### Method

#### Participants

Thiry-one undergraduate students (26 females and 5 males) participated in the experiment, with an average age of 20.23 ± 0.92 years (18–22 years). Four of them were left-handed and others were right-handed. The other characteristics were the same as those of the pre-experiment. The calculated sample size of the pre-experiment used by G * Power was 15, therefore, the amount of participants is sufficient.

#### Experimental instruments, materials, procedures and design

Different from Experiment 1, the non-target boxes in the target display would not turn red or green but would be replaced by purple (RGB: 255 0255; see Figure 1). The other features were the same as those of experiment 1.

### Results and analysis

**Figure 5:**
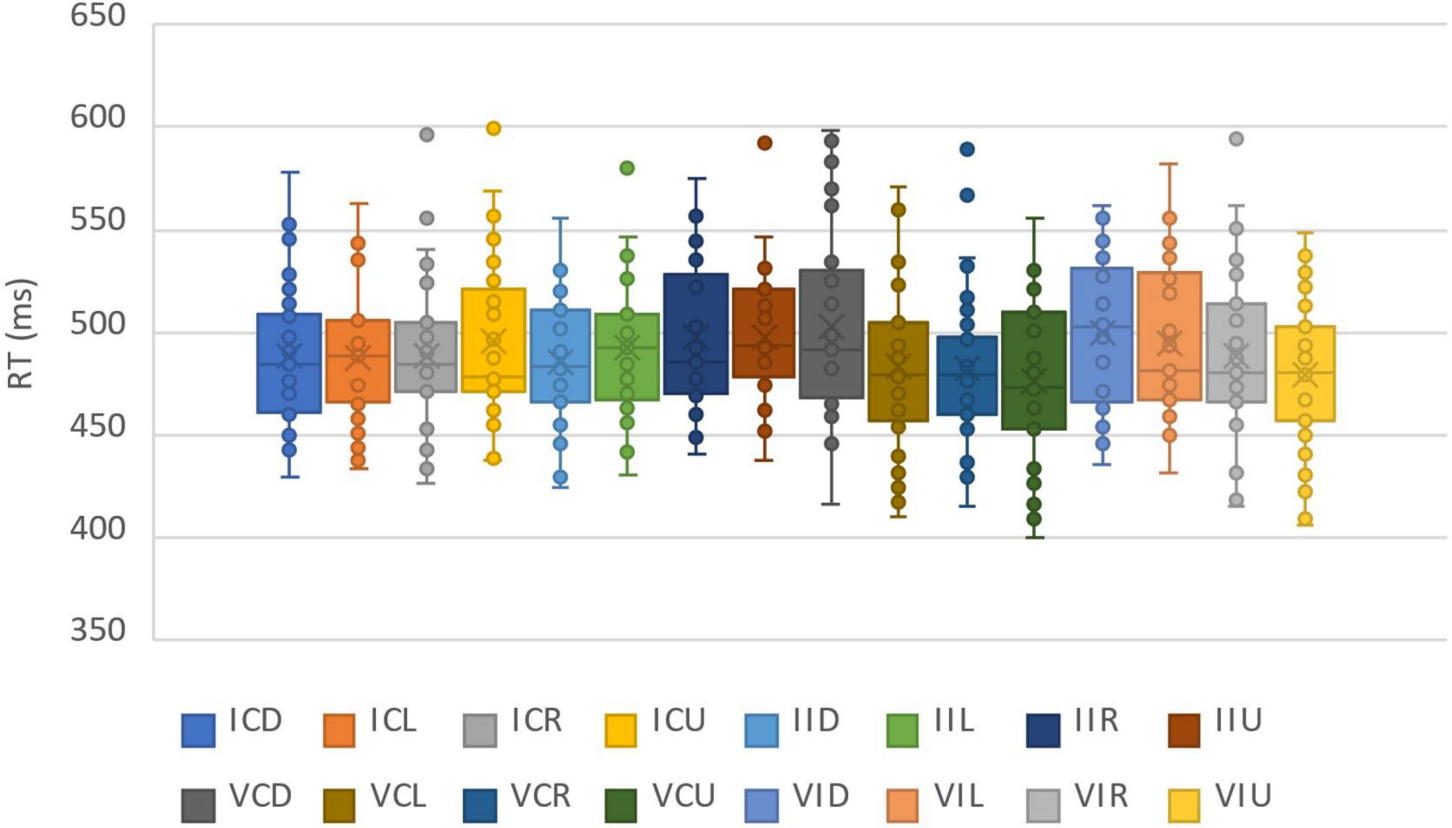
The data distribution of all participants under all conditions in Experiment 2. The description of the legend is the same as that of Experiment 1.

**Figure 6:**
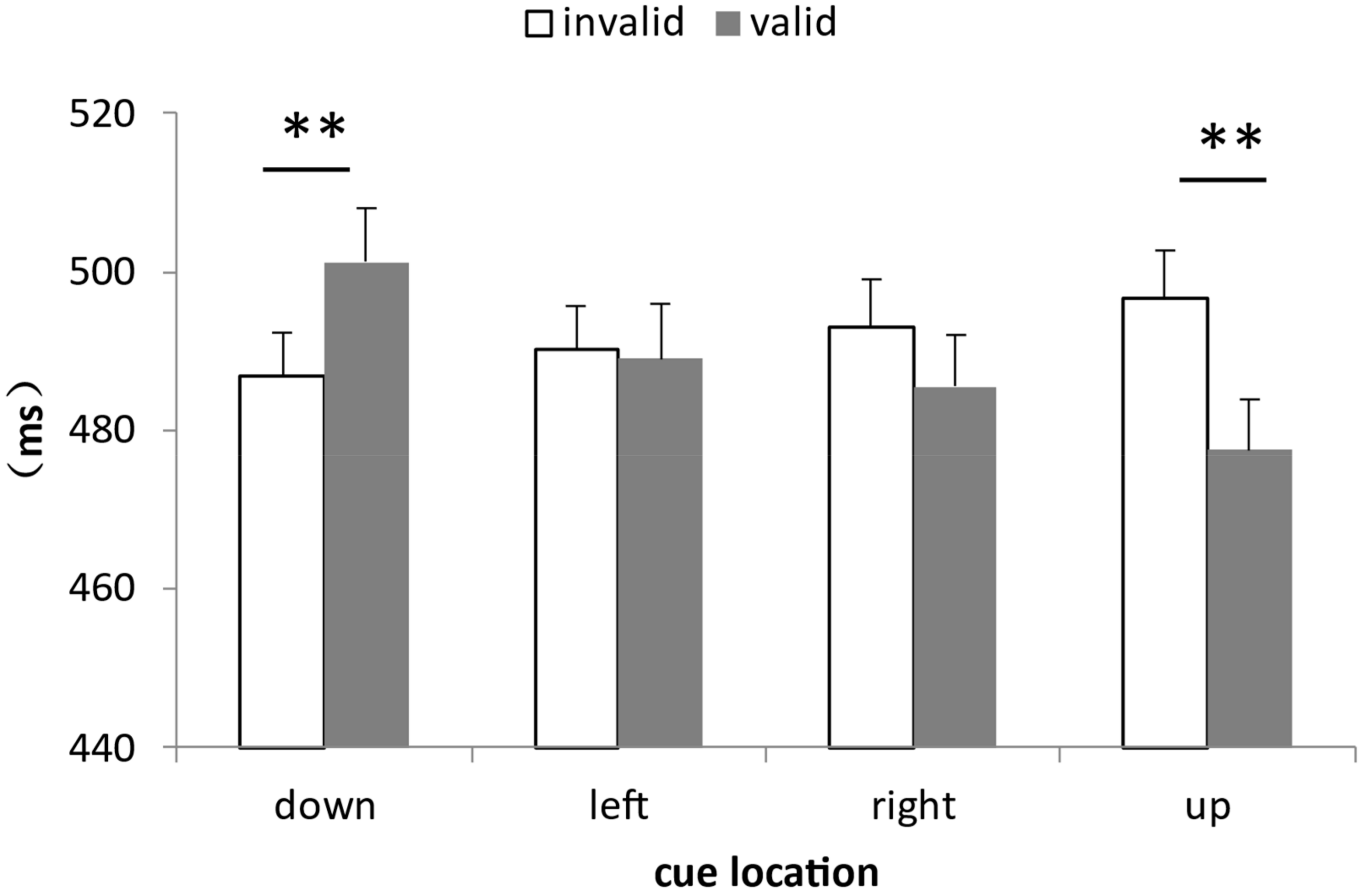
Interaction between cue validity and cue location in Experiment 2. When the cue appeared in the lower position, the response when the cue was invalid was faster than that when the cue was valid; when the cue appeared in the upper position, the response when the cue was invalid was slower than that when the cue was valid; and when the cue appeared on the left and right sides, there was no difference between the response when the cue was invalid and the response when the cue was valid (**p < 0.01).

For the analysis of RTs, a total of 7.42% of the data were deleted by deleting the records of incorrect reactions and the records of those reaction times outside the three standard deviations above and below the average. The data distribution of all participants under all conditions was shown in Figure 5. We calculated the average response times and accuracy rates under the combinations of the cue validity, the cue location and the cue-target semantic congruency (Table 3). The repeated measure ANOVA of response time showed that the main effects of cue validity and cue-target semantic congruency were not significant, which were *F*(1,30) = 1.53, *p* = 0.23 and *F*(1,30) = 1.44, *p* = 0.24, respectively. The main effect of cue location was significant, *F*(3,90) = 4.38, *η_p_^2^* = 0.13, *p* = 0.006. Multiple comparisons showed that the lower position (494.02 ms) was significantly different from the right position (489.25 ms) and the upper position (487.07 ms), *p* = 0.006 and *p* < 0.001, respectively. The interaction of the three factors was not significant, *F*(3,90) = 0.69, *p* = 0.56. More importantly, the interaction between cue validity and cue location was significant, *F*(3,90) = 13.16, *η_p_^2^* = 0.31, *p* < 0.001. A simple effect test showed that when the cue appeared in the lower position, the response when the cue was invalid (486.78 ms) was faster than that when the cue was valid (501.27 ms), *F*(1,30) = 10.01, *η_p_^2^* = 0.25, *p* = 0.004. When the cue appeared in the upper position, the response when the cue was invalid (496.56 ms) was slower than that when the cue was valid (477.58 ms), *F*(1,30) = 23.14, *η_p_^2^* = 0.44, *p* < 0.001. When the cue appeared on the left and right sides, there were no differences between the response when the cue was invalid and the response when the cue was valid, which were *F*(1,30) = 0.06, *p* = 0.81 and *F*(1,30) = 3.88, *p* = 0.06, respectively (see Figure 6). The interaction between semantic congruency and cue location was significant, *F*(3,90) = 3.76, *η_p_^2^* = 0.11, *p* = 0.01. The simple effect test showed that when the cue appeared in the left position, the response of semantic consistency (485.66 ms) was faster than in the case of semantic inconsistency (493.60 ms), *F*(1,30) = 4.63, *η_p_^2^* = 0.13, *p* = 0.04. When the cue appeared in other positions, there were no differences between the responses of semantic congruency and the responses of semantic incongruency, *F*s ≤ 2.67, *p*s ≥ 0.11. The interaction between semantic congruency and cue validity was not significant, *F*(1,30) = 0.30, *p* = 0.56. At the same time, we also analyzed the interactions among the target position and other factors and found that there were no two-factor or three-factor interactions among the target position, semantic congruency and cue location, *F*s ≤ 2.12, *p*s ≥ 0.08.

**Table 3:** The average response times and accuracy rates under the combinations of cue validity, cue-target semantic congruency and cue location in Experiment 2 (M ± SD).

	Congruency	Cue validity	Cue location			
			Down	Left	Right	Up
RT (ms)	Congruent	Invalid	488.17 ± 34.90	487.92 ± 32.45	488.88 ± 37.61	495.56 ± 38.67
		Valid	502.61 ± 45.37	483.39 ± 43.83	482.28 ± 38.60	476.21 ± 37.61
	Incongruent	Invalid	485.39 ± 30.91	492.47 ± 33.11	497.18 ± 34.76	497.57 ± 31.86
		Valid	499.92 ± 35.33	494.73 ± 37.37	488.66 ± 41.86	478.95 ± 36.81
ACC (%)	Congruent	Invalid	92.26 ± 6.02	94.06 ± 5.45	92.68 ± 5.36	92.16 ± 5.78
		Valid	94.29 ± 7.54	89.42 ± 12.80	90.94 ± 11.35	96.42 ± 5.81
	Incongruent	Invalid	91.74 ± 5.31	93.29 ± 5.18	93.29 ± 4.87	91.19 ± 6.66
		Valid	93.39 ± 7.23	90.77 ± 11.36	90.26 ± 9.24	96.84 ± 4.99

In addition, the repeated measurement ANOVA of accuracy showed that the main effects of cue validity and cue-target semantic congruency were not significant, which were *F*(1,30) = 0.08, *p* = 0.78 and *F*(1,30) = 0.11, *p* = 0.74, respectively. The main effect of cue location was significant, *F*(3,90) = 3.34, *η_p_^2^* = 0.10, *p* = 0.02. Multiple comparisons showed that the accuracy of the upper position was higher than that of other positions, *p*s ≤ 0.02. The interaction of three factors was not significant, *F*(3, 90) = 0.73, *p* = 0.53. The interaction between cue validity and cue location was significant, *F* (3, 90) = 8.28, *η_p_^2^* = 0.22, *p* < 0.001. The simple effect test showed that when the cue appeared in the upper position, the correct rate when the cue was invalid (91.68%) was lower than that when the cue was valid (96.93%), *F* (1, 30) = 16.48, *η_p_^2^* = 0.36, *p* < 0.001. When the cue appeared in other places, there were no differences between the correct rate when the cue was invalid and the correct rate when the cue was valid, *F*s ≤ 4.09, *p*s ≥ 0.052. The interactions between the other two factors were not significant. They were the interaction between semantic congruency and cue validity, *F*(1,30) = 0.19, *p* = 0.66 and the interaction between semantic congruency and cue location, *F*(3,90) = 0.20, *p* = 0.90. Through the calculation of G*power software, the statistical power of the significant results of the experiment was greater than 0.95. At the same time, participants reported subjectively that they were unable to perceive or identify the cues.

### Discussion

In Experiment 2, after excluding the attentional control setting based on the displaywide features, it was found that the subliminal cues could still trigger the spatial attentional orientation effects, which were manifested in the inhibition effect in the lower visual field, the capture effect in the upper visual field, and the lack of effect in the left and right visual fields. Comparing the attentional orientation effects obtained in Experiment 1 and Experiment 2, the independent sample t test found that for the lower position, the inhibitory effects obtained in the two experiments were consistent (−19.08 ms and − 14.49 ms), *t*(60) = −0.65, *p* = 0.52, and for the upper position, the capture effects of the two experiments were also consistent (26.10 ms and 18.98 ms), *t*(60) = 1.09, *p* = 0.28. The difference between Experiment 1 and Experiment 2 was mainly reflected in the left and right positions, which was also the result of the attentional control setting based on displaywide features. It could be seen that the attentional orientation effect affected by the setting based on the displaywide features was reflected in the space on the left and right sides of the horizontal, and the effect size was about 15 ms (the average value of the difference between the effect sizes on the left and right sides of the two experiments), which was the same on the left and right sides. Therefore, at least part of the attentional orientation effect found in Experiment 1 came from the attentional orientation based on the attentional control setting based on displaywide features, and its effect was reflected in the left and right spatial positions. In addition, the experiment found that when the cues appeared on the left, the response when the cue-target was semantically congruent was faster than that when the cue-target was semantically inconsistent. We consider that this is a semantic-masking priming effect, that is, the subliminal masked stimulus can promote the processing of subsequent targets through semantic priming, indicating that under certain conditions, the semantic analysis of cues does not need conscious participation (Dehaene et al., 1998; Jaśkowski et al., 2002). Some studies have found that subliminal masking semantic priming is affected by top-down control and depends on the availability of current attentional resources. When the available attentional resources are reduced, the degree of subliminal masking semantic priming will also decrease (Martens and Kiefer, 2009). In Experiment 1, no subliminal masking semantic priming effect was found. In Experiment 2, the subliminal masking semantic priming effect only appeared in some places. This may be due to the change in task conditions, which makes the top-down control different in the two experiments, resulting in the difference in available attentional resources.

Then, after excluding the attentional control setting based on displaywide features, the attentional orientation effect found in Experiment 2 may come from two aspects, one is the attentional control setting based on general attributes, and the other is the abrupt onset formed by the subliminal cue and it subsequent mask.

## Experiment 3: The attentional orientation excluding the attentional control setting based on general attributes

In order to separate the effects of the attentional control setting based on general attributes and the abrupt onset of the subliminal cue and its mask on the spatial attentional orientation of subliminal cues in Experiment 2, Experiment 3 eliminated the meaning of subthreshold cues so that there was no meaningful contingency between cues and targets. The results of the experiment should reflect the role of the abrupt onset of subliminal cues and their masks. The experimental hypothesis is that if the results of Experiment 2 and Experiment 3 are consistent, the attentional control setting based on general attributes does not play a role in subliminal meaning-contingent attentional orientation, and the results are derived from the abrupt onset of the subliminal cues and their masks; however, if the results of the two experiments are different, it indicates that the attentional control setting based on the general attributes played a role and the effect is the difference between the two experiments.

### Method

#### Participants

Twenty-six undergraduate students (21 females and 5 males) participated in the experiment, with an average age of 19.82 ± 1.21 years (18–23 years). One of them was left-handed and others were right-handed. The other characteristics were the same as those in the pre-experiment. The calculated sample size of the pre-experiment used by G * Power was 23, therefore, the amount of participants is sufficient.

#### Experimental instruments, materials, procedures and design

There was only one kind of cue in Experiment 3, that was the white Chinese character “纱” without any color meaning (see Figure 1). A 2× 4 repeated measurement design was used. The first independent variable was cue validity, including two levels: cue valid and cue invalid. The second independent variable was the cue location, which included four levels: up, down, left and right. The experiment lasted about 20 min. The other features were the same as those of Experiment 2.

### Results and analysis

**Figure 7:**
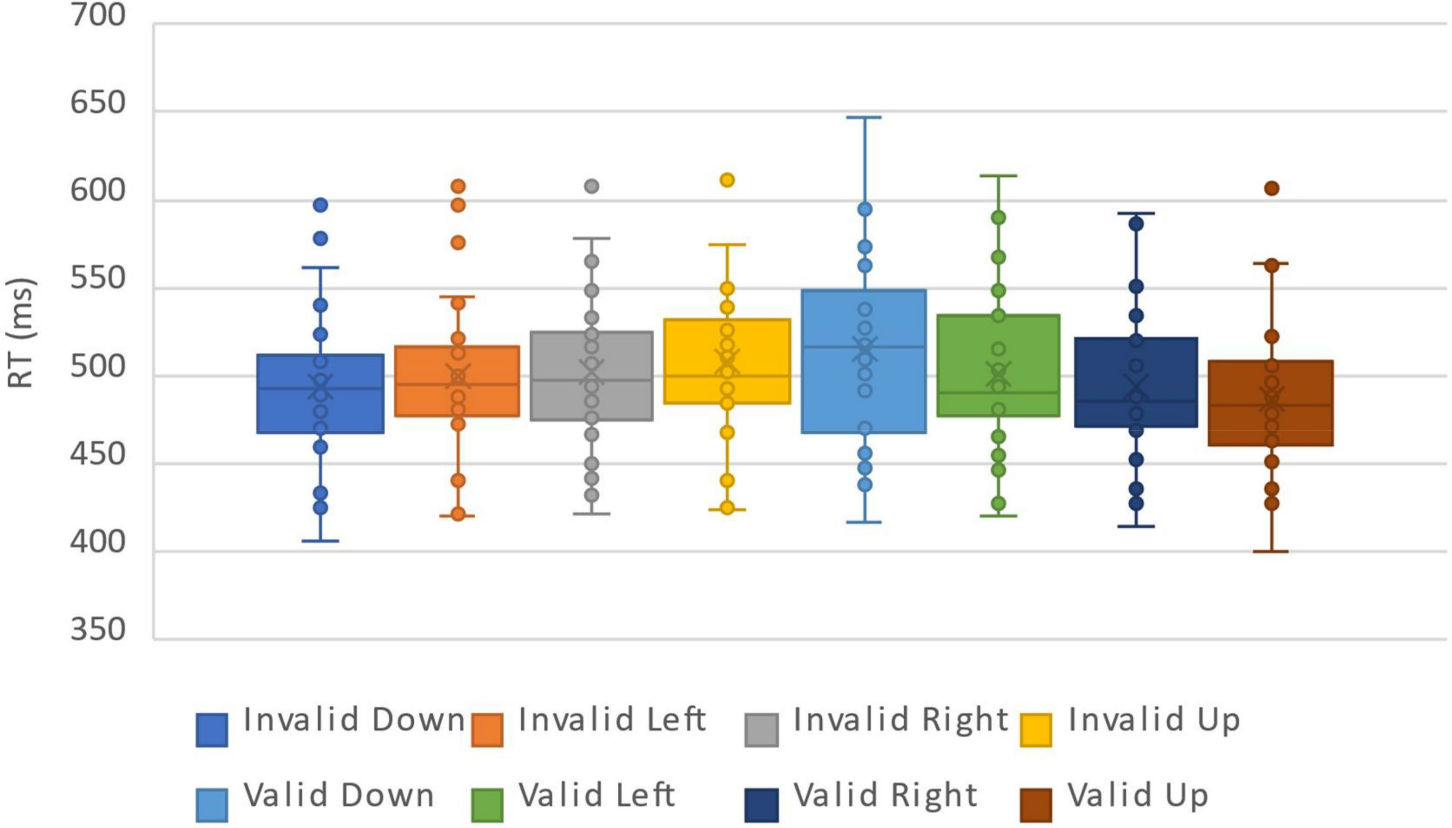
The data distribution of all participants under all conditions in Experiment 3.

**Figure 8:**
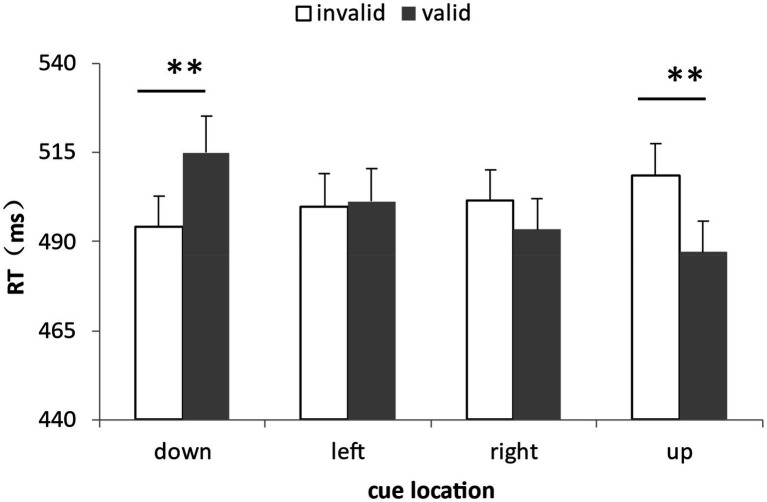
Interaction between cue validity and cue location in Experiment 3. When the cue appeared in the lower position, the response when the cue was invalid was faster than that when the cue was valid; when the cue appeared in the upper position, the response when the cue was invalid was slower than that when the cue was valid; and when the cue appeared on the left and right side, there was no difference between the response when the cue was invalid and the response when the cue was valid (**p < 0.01).

For the analysis of RTs, a total of 8.72% of the data were deleted by deleting the records of incorrect reactions and the records of those reaction times outside the three standard deviations above and below the average. The data distribution of all participants under all conditions was shown in Figure 7. We calculated the average response times and accuracy rates under the combinations of cue validity and cue location (Table 4). The repeated measure ANOVA of response time showed that the main effects of the two factors were not significant: cue effectiveness, *F*(1,25) = 0.27, *p* = 0.61; and cue location, *F*(3,75) = 2.01, *p* = 0.12, respectively. The interaction of the two factors was significant, *F*(3,75) = 13.65, *η_p_^2^* = 0.35, *p* < 0.001. The simple effect test showed that when the cue appeared in the lower position, the response when the cue was invalid was faster than that when the cue was valid, *F*(1,25) = 9.21, *η_p_^2^* = 0.27, *p* = 0.006. When the cue appeared in the upper position, the response when the cue was invalid was slower than that when the cue was valid, *F*(1,25) = 14.97, *η_p_^2^* = 0.37, *p* = 0.001. When the cue appeared on the left and right sides, there was no difference between the response when the cue was invalid and the response when the cue was valid, which were: left position, *F*(1,25) = 0.10, *p* = 0.76, and right position, *F*(1,25) = 2.56, *p* = 0.12 (see Figure 8). At the same time, there was no interaction between target location and cue location, *F*(9, 25) = 0.48, *p* = 0.89.

**Table 4:** The average response times and accuracy rates of the combinations of cue validity and cue location in Experiment 3 (M ± SD).

	Cue validity	Cue location			
		下	左	右	上
RT (ms)	Invalid	494.25 ± 43.33	499.94 ± 46.61	501.51 ± 44.04	508.42 ± 47.16
	Valid	514.90 ± 53.29	501.28 ± 47.01	493.32 ± 44.92	487.03 ± 44.51
ACC (%)	Invalid	91.00 ± 6.28	92.88 ± 4.83	90.88 ± 8.49	90.15 ± 7.10
	Valid	90.58 ± 11.23	90.73 ± 10.04	91.38 ± 6.74	92.96 ± 7.67

In addition, the repeated measurement ANOVA of accuracy showed that the main effects of the two factors were not significant, which were: cue effectiveness, *F*(1,25) = 0.05, *p* = 0.83 and cue location, *F*(3,75) = 0.30, *p* = 0.83. The interaction of the two factors was not significant, *F*(3,75) = 1.12, *p* = 0.35. Through the calculation of G*power software, the statistical power of the significant results of the experiment was greater than 0.95. At the same time, participants reported subjectively that they were unable to perceive or identify the cues.

### Discussion

Experiment 3 excluded the meaningful contingency between cue and target. At the same time, the target in Experiment 3 was formed by changing the color on the basis of the original boxes and did not have the attribute of abrupt onset. Therefore, it was not contingent on the subliminal abrupt onset cues. The results of the experiment should be attributed to the bottom-up processing of abrupt onsets. The results showed that there were inhibition and capture effects in the lower and upper positions, respectively, and there were no attentional orientation effects in the left and right positions. For the inhibition effect of the lower position, Experiment 3 (−20.64 ms) and Experiment 2 (−14.49 ms) were consistent, *t*(55) = 0.77, *p* = 0.44. For the capture effect of the upper position, Experiment 3 (21.39 ms) and Experiment 2 (18.98 ms) were consistent, *t*(55) = −0.36, *p* = 0.72. It can be seen that the results of Experiment 2 and Experiment 3 are consistent; that is, the attentional orientation effect found in Experiment 2 is contributed by the abrupt onset of subliminal cues and their masks, and the attentional control setting based on general attributes has no effect, while the bottom-up attentional orientation effect caused by subliminal cues and their masks is mainly manifested in the upper and lower visual fields with opposite polarity.

**Figure 9:**
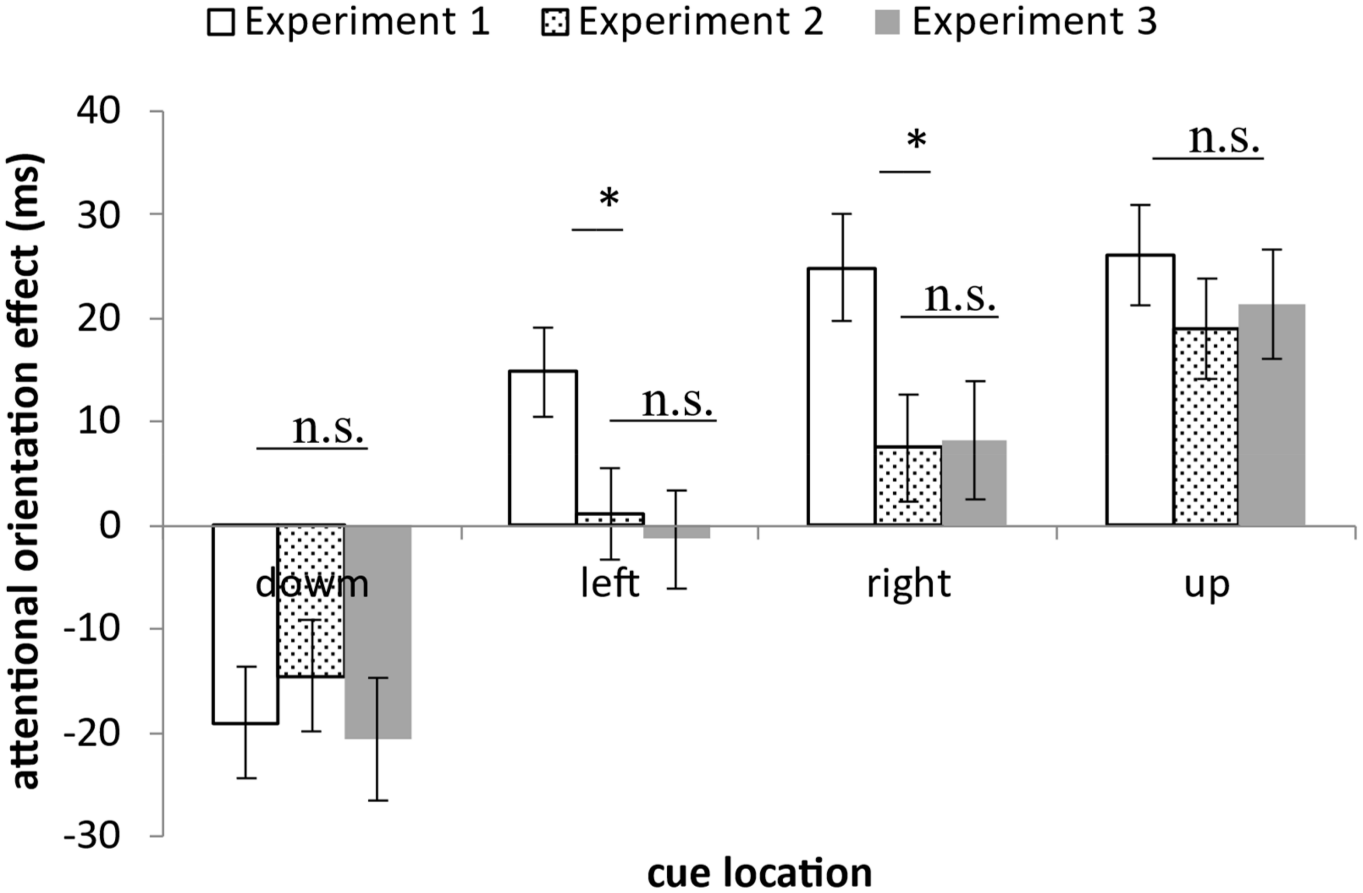
Attention orientation effects under different cue positions in Experiments 1–3. When the subliminal cue appeared at the lower and upper positions, there was no difference in the attentional orientation effects among the experiments. While when the subliminal cue appeared at the left and right positions, there was a significant difference in attentional orientation effects among the experiments. Specifically, the attentional orientation effect in Experiment 1 was significantly different from that in Experiment 2 and Experiment 3, while there was no significant difference in attentional orientation effect between Experiment 2 and Experiment 3 (*p < 0.05, n.s. not significant).

In order to compare the attentional orientation effects found in the three formal experiments in this study, we comprehensively compared the attentional orientation effects of Experiments 1–3 under each cue position. It was found that when the subliminal cue appeared at the lower and upper positions, there was no difference in the attentional orientation effects among the experiments, *F*s ≤ 0.56, *p*s ≥ 0.57. When the subliminal cue appeared at the left and right positions, there was a significant difference in attentional orientation effects among the experiments, *F*s ≥ 3.52, *p*s ≤ 0.03. Specifically, the attentional orientation effect in Experiment 1 was significantly different from that in Experiment 2 and Experiment 3, *p*s ≤ 0.03, while there was no significant difference in attentional orientation effect between Experiment 2 and Experiment 3, *p*s ≥ 0.71 (see Figure 9). From these results, it can be found that the attentional orientation effect found in Experiment 1 should be composed of two parts. The first is the top-down effect caused by the attentional control setting based on the displaywide features, which is manifested on the left and right sides; The second part is the bottom-up effect caused by the abrupt onset of subliminal cues and their masks, which is manifested in the upper and lower positions.

## General discussion

In this study, one pre-experiment and three formal experiments were conducted to explore the subliminal meaning-contingent attentional orientation by using the cueing paradigm and backward masking and analyzed its composition. Through the chance level response to the masked cues and the subjective reports of participants, the pre-experiment determined that the cues in this study had reached the subliminal level, and their meaning could not be perceived. In Experiment 1, the meaning contingency between objects was established in the form of Chinese character cues and color targets. The results showed the inhibition effect and the capture effects in the other fields. In order to separate the attentional control setting used in Experiment 1, Experiment 2 excluded the meaning contingency between cues and non-target objects in the target display, that was the attentional control setting based on the displaywide features, and found the inhibition effect in the lower visual field and the capture effect in the upper visual field which were consistent with Experiment 1, while the capture effects of the left and right sides in Experiment 1 disappeared, indicating that the attentional control setting based on the displaywide features in Experiment 1 had an effect, which was reflected in the left and right positions that showed the equal orientation in the left and right visual fields. In order to clarify the effects of the general attributes-based attentional control setting and the abrupt onset formed from the subliminal cues and their masks in Experiment 2, Experiment 3 excluded the possible meaning contingency between cues and targets, and found the results consistent with Experiment 2, which showed that the attentional orientation effect in Experiment 2 was the result of the abrupt onset from subliminal cues and their masks, while the general attributes-based attentional control setting did not play a role.

This study extends the applicability of the contingent attentional orientation hypothesis; that is, the contingent attentional orientation can occur not only at the level of abstract meaning, but also under the subliminal condition of imperceptible meaning. This study uses backward masking to make the cue meaning imperceptible, and uses the experimental design consistent with the supraliminal meaning-contingent attentional orientation study (Wang et al., 2021). We obtained relatively consistent results that suggest even if the cue meaning is imperceptible, the meaning contingency between objects can still modulate the visual spatial attention. This result shows that when there is meaning contingency between objects, even if there is no conscious processing, the object’s meaning can still modulate the spatial attention allocation according to the current attentional control setting, and the meaning-contingent attentional orientation can occur at the subliminal level and is not affected by consciousness. It is worth noting that although the test results of subliminal cue visibility in some studies were higher than the chance level, it was still considered that the cue met the subliminal requirements (Weichselbaum et al., 2014; Schoeberl et al., 2015). Prasad and Mishra (2020) posited that there were several reasons: the first, the participants reported subjectively that they were not aware of the cues; the second, the participants in the visibility test task needed to pay attention to the cues according to the task requirements, so the visibility index of the cues in the test task was overestimated; and finally, the effect of the subliminal cue itself has been shown in the participants’ responses. In the pre-experiment of this study, the response to cues was by chance, and the participants reported subjectively that they could not perceive the cues, so the subliminal state of cues in this study can be guaranteed. In the formal experiments, there was no requirement to pay attention to the cues, and the cues were not predictive. Therefore, participants have no need to pay attention to the cues; that is, they have no subjective intention to pay attention to the cues. Therefore, the cue effects obtained in the experiments are the result of involuntary attention.

As for the influence of consciousness perception on attentional orientation, some researchers compared the attentional orientation processing with and without consciousness by manipulating the participants’ consciousness of cues (Giattino et al., 2018). In terms of behavioral results, the cue effects were observed both in conscious and unconscious conditions, but the former was more obvious than the latter. In terms of electrophysiological results, the N2pc (N2 posterior contralateral) component appeared only in the conscious condition. N2pc is the contralateral negative potential difference in the posterior part of the cortex 180–300 ms after the presentation of the stimulus, which is considered to reflect the selection and allocation of attention resources to this object (Luck and Hillyard, 1994). Although the N2pc component did not appear in the unconscious condition, when the cue was valid, the P1 component when the cue was valid was stronger than P1 when the cue was invalid. P1 is a sensory component, which is related to the feedforward visual processing of the low-level cortex (Luck and Kappenman, 2011), which indicated that although the subliminal cue was not detected, the target appearing in its position still got strong sensory processing. These results suggested that attention could be directed to the subliminal cues and led to an enhanced sensation of subsequent stimuli. Different from the neural mechanism of perceptual stimulus processing, unconscious stimulus processing may be through the subcortical pathway. Recently, some researchers have investigated the relationship between consciousness and attention (Baier et al., 2020). For these two independent variables, the visibility or consciousness of stimulus was manipulated by changing the time interval between the stimulus and the mask, and the stimulus-driven attentional capture was manipulated by changing the significance of the stimulus in all objects. The results showed that whether the stimulus was visible or not, the singleton led to the phenomenon of attentional capture, or the stimulus-mask time interval (consciousness) and the manipulation of the singleton (attention) affected the results in an independent way. Therefore, researchers believed that stimulus-driven attention and consciousness are independent of each other, and stimulus-driven attentional capture can occur before consciousness. Some researchers believed that perceptual consciousness depends on spatial attention. Spatial attention is a prerequisite for internal representation of space, which provides a medium for perceptual experience. Spatial attention is necessary for the internal representation of space, without which the perceptual awareness of stimuli cannot occur (Koivisto et al., 2009). Our study found that when the cue is imperceptible, it can also modulate the spatial attention according to the current task, which means that attention can point to subliminal stimuli, and there is no need for conscious participation in this process. Therefore, for the relationship between the two, some researchers have concluded that attention is a necessary but insufficient condition for conscious perception (Sean and Mangun, 2020).

In Experiment 1 of this study, the meaning-contingent attentional orientation of the subliminal cues was found. Because the attentional orientation effect was not affected by the cue-target semantic congruency, participants did not use the attentional control setting based on the specific features or concepts. Through the comparison between Experiment 1 and Experiment 2, it can be seen that the attentional control setting based on the displaywide features has an effect on spatial attentional orientation, which is manifested in the capture effects of the horizontal left and right positions with the same polarity and the same size. Through the comparison between Experiment 2 and Experiment 3, we can see that the attentional control setting based on general attributes has no effect on spatial attentional orientation. For the perceptual contingent attentional orientation, some researchers believed that the subliminal cues can capture attention in a goal-driven manner, indicating that top-down factors can affect subliminal spatial attentional orientation. For example, in the study of Ansorge et al. (2009), the participants searched for a target of a certain color, the target appeared in half of the trials but not in the other half of the trials. The results showed that subliminal cue that did not predict the position of the target but was consistent with the color of the target not only caused the spatial cue effect in behavior, but also triggered the N2pc component representing attentional selection, even if the target was not present in that trial. According to these results, the researchers argued that the attentional capture influenced by task setting could be triggered not only by supraliminal stimuli, but also by subliminal stimuli that were not perceived. Subsequently, Ansorge et al. (2010) repeated the main results of Ansorge et al. (2009) and added that when the colors of the cue and the target were inconsistent, the cue of the same intensity could not lead to the cue effect, nor could it trigger the N2pc component. Recently, Travis et al. (2019) not only found the N2pc component of the cue that was consistent with the target color, but also found P_D_ component that was inconsistent with the target color but consistent with the distractor color. P_D_ is a positive component, representing the inhibitory processing of distractors (Sawaki and luck, 2010). The experimental results of Travis et al. (2019) showed that whether the cue was perceived or not, it triggered the corresponding attentional selection enhancement and inhibition effects, but the N2pc component in conscious conditions was stronger than the N2pc component in the unconscious condition, and there was no difference in the P_D_ component in the two conditions. Therefore, researchers considered that the attention selection enhancement of visual features was modulated by conscious perception, while the inhibition of visual features might be independent of consciousness.

Some researchers have discussed how the selective processing of unconscious stimuli contingent on current task goals is achieved in the brain (Ulrich et al., 2014). In their research, the task setting based on the semantic or perceptual level was established respectively, and then the subliminally primed lexical decision task was carried out. The results showed that compared with the task setting at the perceptual level, the brain regions responsible for semantic processing were more activated under task settings at the semantic level, which also enhanced the processing of semantic attributes of unconscious stimuli. The researchers believed that unconscious processing depended on the attentional enhancement of the processing path contingent on the task, which was achieved through the dynamic modulation of functional communication in the brain. In the attentional control of unconscious processing, it was an important processing process to dynamically establish the functional network contingent on the task setting. As far as we know, our study is the first study on subliminal meaning-contingent attentional orientation. From the results, different kinds of attentional control settings may have different roles in spatial attentional orientation. At least in this study, the attentional control setting based on the specific features and the attentional control setting based on non-specific general attributes had not played a role. All the top-down spatial attention processing was contributed by the attentional control setting based on the displaywide feature and only appeared in the horizontal positions, with the limitation of spatial position. This study found that cue validity was not restricted by the cue-target semantic congruency, which was consistent with the results of Wang et al. (2021), indicating that participants did not adopt the semantically contingent attentional control setting based on specific features. In a study of a similar experimental design, Wang et al. (2018) found that the same cue did not cause the spatial cueing effect when the cue and target were semantically inconsistent, indicating that participants adopted the semantically contingent attentional control setting based on specific features. This seems to indicate that the standard of which attentional control setting is adopted in which situation is flexible, and the applicability of various attentional control settings in spatial attentional orientation needs to be further investigated.

## Conclusion

The meaning-contingent attentional orientation can occur at the subliminal level, which is not restricted by conscious perception. In particular, the attentional control setting based on the displaywide features plays a role in the subliminal meaning-contingent attentional orientation, which shows the same capture effects on the left and right sides of the horizontal. This study refined and separated the spatial attentional orientation effect, supported the contingent involuntary attentional orientation hypothesis, and expanded its scope of application.

## Data availability statement

The datasets presented in this study can be found in online repositories. The names of the repository/repositories and accession number(s) can be found in the article/Supplementary material.

## Ethics statement

The studies involving human participants were reviewed and approved by School of Philosophy and Sociology of Jilin University. The patients/participants provided their written informed consent to participate in this study.

## Author contributions

HW participated in the experimental design, data processing, and manuscript writing. JY and YG participated in the experimental design and discussion. MZ participated in the revision and polishing of the manuscript. All authors contributed to the article and approved the submitted version.

## Funding

This work was supported by a grant from the Humanistic and Social Sciences Research Project of the Education Department of Jilin Province, China (No. JJKH20220806SK) and the Scientific Research Program of Jilin Provincial Education Society during the “Fourteenth Five Year Plan”, China (No. G210488) to HW, JST FOREST Program (No. JPM-JFR2041) to JY, the Humanity and Social Science Youth Foundation of Ministry of Education of China (22YJC190005), Scientific Research Project of the Education Department of Jilin Province, China (No. JJKH20211101KJ) and Fundamental Research Funds for the Central Universities (No. 2021ZZ028) to YG, and National Nature Science Foundation of China (No. 31700939) and Japan Society for the Promotion of Science (No. 20K04381) to MZ.

## Conflict of interest

The authors declare that the research was conducted in the absence of any commercial or financial relationships that could be construed as a potential conflict of interest.

## Publisher’s note

All claims expressed in this article are solely those of the authors and do not necessarily represent those of their affiliated organizations, or those of the publisher, the editors and the reviewers. Any product that may be evaluated in this article, or claim that may be made by its manufacturer, is not guaranteed or endorsed by the publisher.

## Footnotes

^1^It is suggested that gender does not affect visual spatial attention (Inukai and Kawahara, 2012). The same below.
